# rfaG-dependent cell envelope biogenesis supports type 1 fimbrial expression and intestinal colonization in adherent-invasive Escherichia coli

**DOI:** 10.1099/mic.0.001766

**Published:** 2026-09-02

**Authors:** Tsuyoshi Miki, Hana Yamaguchi, Takeshi Haneda, Masahiro Ito, Yun-Gi Kim

**Affiliations:** 1 Department of Microbiology, School of Pharmacy, Tohoku Medical and Pharmaceutical University, Sendai, Miyagi, Japan; 2 Department of Microbiology, School of Pharmacy, Kitasato University, Tokyo, Japan

**Keywords:** adherent-invasive *Escherichia coli*, epithelial invasion, intestinal colonization, *rfaG-*dependent cell envelope biogenesis, type 1 fimbriae

## Abstract

Increased intestinal colonization by adherent-invasive *Escherichia coli* (AIEC) has been implicated in Crohn’s disease, an inflammatory bowel disease characterized by abnormal immune responses and chronic inflammation in the gastrointestinal tract. LPS–Toll-like receptor 4 signalling may contribute to AIEC-associated intestinal inflammation. Here, we show that disruption of *rfaG*-dependent cell envelope biogenesis is associated with reduced type 1 fimbrial expression, epithelial invasion and intestinal colonization in the AIEC strain LF82. Deletion of *rfaG*, a gene involved in cell envelope biogenesis, resulted in reduced type 1 fimbrial expression and activity, accompanied by reduced transcription of *fim* genes. The *rfaG* mutant also exhibited impaired invasion of epithelial cells compared with the parental LF82 strain. Finally, the *rfaG* mutant showed reduced competitive colonization in the murine intestinal tract, whereas chromosomal complementation with *rfaG* restored this colonization defect. Inducible expression of type 1 fimbriae also partially reversed the reduced colonization at the early stage of infection, raising the possibility that additional *rfaG*-dependent mechanisms, independently of type 1 fimbrial expression, contribute to intestinal colonization. These findings suggest that *rfaG*-dependent cell envelope biogenesis contributes to the coordinated expression of surface-associated virulence factors associated with intestinal colonization in AIEC.

## Introduction

LPS constitutes the outer leaflet of the outer membrane bilayer of Gram-negative bacteria and contributes to diverse physiological processes including structural integrity, motility and attachment and invasion of host cells. LPS is a tripartite molecule composed of lipid A, a core oligosaccharide and O-antigen [1]. Lipid A is highly conserved and confers endotoxic activity that induces proinflammatory responses [2]. The core oligosaccharide is relatively conserved; *Escherichia coli* strains possess five major core types: K-12, R1, R2, R3 and R4 [3]. Genes involved in core biosynthesis are clustered in the *rfa* locus. Mutations in genes involved in LPS core biosynthesis can generate rough mutants that produce truncated LPS, although the resulting LPS structure depends on the specific gene affected [4, 5]. For instance, an *E. coli rfaG* mutant with LPS truncated after HepII has compromised outer membrane barrier function due to the complete lack of phosphate groups on HepII and only 40% phosphate substitution at HepI [6]. In contrast, O-antigen is a highly variable polysaccharide chain, as reflected by the presence of more than 180 different O serotypes in *E. coli* [1, 7, 8]. O-antigen is linked to serotype specificity and contributes to bacterial resistance to complement-mediated lysis [9]. In addition to its protective role as part of the outer membrane barrier, O-antigen influences envelope-associated properties including fimbriae production and flagellar function [10, 11].

Adherent-invasive *E. coli* (AIEC) strain LF82 was originally isolated from an ileal lesion of a patient with Crohn’s disease (CD) [12], an inflammatory bowel disease characterized by chronic inflammation and severe injury of the gastrointestinal tract [13]. Although LF82 has been extensively used as the prototypical AIEC strain, subsequent studies have demonstrated considerable genetic and phylogenetic diversity among AIEC isolates including NRG857c, HM605, UM146 and other clinical strains, suggesting that the AIEC phenotype has evolved through multiple independent genetic pathways rather than a single lineage. Nevertheless, LF82 remains one of the best-characterized AIEC strains and continues to serve as a representative experimental model. Furthermore, recent evidence suggests that SNPs in *rpoD*, which encodes the primary sigma factor (σ^70^), may contribute to the enhanced fitness of LF82 within the intestinal environment of CD patients, although this SNP appears to be specific to LF82 compared with the laboratory strain MG1655 [14]. AIEC strains are significantly more prevalent in patients with CD than in healthy individuals [15, 16], and their association with CD pathogenesis is now widely recognized [17–20]. Accumulating evidence indicates that CD-associated AIEC strains adhere to carcinoembryonic antigen-related cell adhesion molecule 6, which is overexpressed on the intestinal epithelial cells of patients with CD, survive within murine and human macrophages as well as epithelial cells and trigger inflammatory cytokine production in infected macrophages [21–24]. However, although the association between AIEC and CD is well established, whether AIEC is a causal driver of disease or preferentially expands within the CD intestinal environment remains unresolved.

AIEC possesses several pathogenic features, including the ability to adhere to and invade intestinal epithelial cells and to survive and replicate within macrophages. Type 1 fimbriae play a central role in AIEC pathogenesis by mediating bacterial adhesion to epithelial cells, a process closely linked to epithelial invasion and intestinal colonization [22, 25, 26]. Accordingly, expression of type 1 fimbriae is tightly regulated through multiple environmental sensing and regulatory pathways [27]. Previous studies have shown that two-component signal transduction systems, including UvrY/BarA, CpxRA and BasRS, modulate transcription of the *fim* operon in response to environmental cues [28–30]. Furthermore, the type 1 fimbrial operon is controlled by phase variation, in which phase switching involves the reversible inversion of a short DNA element called the *fimS* switch by site-specific recombination [31, 32]. The *fimS* switch contains a promoter directed towards the fimbrial genes in the ON orientation but not in the OFF orientation [33, 34]. The inversion reactions are catalysed by two recombinases, FimB and FimE [35, 36]. In addition to the regulation of *fim* gene expression, the biosynthesis of type 1 fimbriae requires periplasmic chaperones and outer membrane-located usher proteins [37], suggesting that cell-surface organization may be associated with type 1 fimbrial expression. These observations provide the rationale for investigating whether *rfaG*-dependent cell envelope biogenesis influences the phase-variable expression of type 1 fimbriae in AIEC.

Although the precise aetiology of CD remains unresolved, accumulating evidence indicates that dysregulated interactions between the intestinal microbiota and mucosal immune system play a central role in disease pathogenesis [38]. Interaction of AIEC with the intestinal mucosa can induce inflammatory responses through binding of LPS to Toll-like receptor 4 (TLR4) expressed on intestinal epithelial cells [39–41]. In addition, AIEC enhances TLR4 expression by suppressing let-7b, a microRNA that negatively regulates TLR4 expression [42]. Notably, AIEC-induced intestinal inflammation together with increased T helper 17 responses requires the synergistic effect of LPS, which induces IL-1β production in mononuclear phagocytes [43]. Thus, previous studies have primarily focused on the role of LPS in host inflammatory responses, whereas its contribution to bacterial surface-associated phenotypes, including type 1 fimbrial expression, remains poorly understood.

LPS is also a major structural component of the outer membrane, and thus defects in LPS core biosynthesis alter outer membrane organization and other cell-surface properties [44]. Furthermore, previous studies have linked LPS core structure to fimbrial production in *E. coli*: mutations in *rfaG*, *rfaP* or *galU* resulted in a deep-rough LPS phenotype accompanied by reduced production of type 1 fimbriae [10], whereas LPS core mutations impaired the biosynthesis or surface expression of K99 fimbriae [45]. These observations suggest that proper LPS core assembly is required not only for maintaining outer membrane integrity but also for the normal expression or assembly of fimbrial structures [37, 46, 47]. However, whether LPS core biosynthesis regulates the phase-variable expression of type 1 fimbriae in AIEC remains unknown. In this study, we investigated whether *rfaG*-dependent cell envelope biogenesis influences type 1 fimbrial expression and thereby affects epithelial invasion and intestinal colonization in AIEC.

## Methods

### Bacterial strains and growth conditions

Bacterial strains and plasmids used in this study are listed in Table 1. AIEC strain LF82, originally isolated from a chronic ileal lesion of a CD patient, belongs to *E. coli* serotype O83:H1 [12]. An isogenic *rfaG* deletion mutant was generated by λ Red recombination using a PCR product, as previously described [48]. The template plasmid pKD46-cat was used to generate kanamycin-resistant LF82 mutants [29]. The primers used for construction of the *rfaG* mutant were LF rfaG-red-FW (5′-TCGATAAATTACTTCCCTCCTCCACGACAGGTACGTCGTTGTGTAGGCTGGAGCTGCTTC-3′) and LF rfaG-red-RV (5′-ATCTTTACCGCGCCATAACGTGGCAAACGGCTCTTTAAGTCATATGAATATCCTCCTTAG-3′). Deletion of the *rfaG* gene was verified by PCR amplification using primers flanking the deleted locus. Bacterial strains were cultured in Luria–Bertani (LB) medium at 37 °C with shaking at 160 r.p.m. [28]. Antibiotics were used at the following final concentrations: ampicillin, 100 µg ml^−1^; kanamycin, 50 µg ml^−1^; and chloramphenicol, 5 or 25 µg ml^−1^.

**Table 1.** Strains and plasmids used in this study

Strain or plasmid	Relevant characteristic	Source or reference
**Strains**		
LF82	*E*. *coli* pathobiont isolated from an ileal biopsy sample of a patient with CD	[12]
S815	LF82 ∆*rfaG*	This study
S879	LF82 *∆rfaG* pLDΩKm2-*rfaG*	This study
T693	LF82 *∆fimAICDFGH* (∆*fimA-H*)::*kan*	[29]
S895	LF82 *∆rfaG* pHSG575	This study
S896	LF82 *∆rfaG* pHSG-*fimA-H*	This study
T597	LF82 ∆*fliC*::*kan*	[29]
S800	LF82 ∆*rfaG*::*kan*	This study
**Plasmids**		
pKD46-cat	Lambda Red recombinase plasmid, Ts replicon, containing *cat* gene	[29]
pLDΩKm2	pLD54-derived suicide plasmid	[72]
pLDΩKm2-*rfaG*	pHSG575 containing *rfaG*, expressing RfaG	This study
pHSG575	Low-copy-number expression vector	[73]
pHSG-*fimA-H*	pHSG575 containing *fimA-H*, expressing FimAICDFGH	[29]

### Construction of a *rfaG* complementation

All cloning procedures involving the suicide integration plasmid pLDΩKm2 were performed using *E. coli* DH5αλ*pir* and S17.1λ*pir* strains [49]. A DNA fragment containing *rfaG* together with the 150 bp upstream region was amplified by PCR using the primers LF rfaG-KpnI-FW (5′-AAAGGTACCCATGATTCAATTCTGGGC-3′) and LF rfaG-SphI-RV (5′-AAAGCATGCGCCATTTGAGAAAATAGC-3′). The amplified fragment was cloned into pLDΩKm2 [50] via KpnI and SphI digestion, yielding pLDΩKm2-*rfaG*. The resulting plasmid was introduced into the ∆*rfaG* mutant by conjugation, and chromosomal integrants carrying the complementing construct were selected on LB agar plates containing ampicillin and kanamycin. Accordingly, a single copy of the pLDΩKm2-*rfaG* construct was integrated into the chromosome to generate the complemented strain.

### Bacterial agglutination assay using O83 antisera

Bacterial agglutination assays using O83 antisera were performed according to the method described by Ørskov and Ørskov [51]. Briefly, bacteria grown in LB medium were boiled at 100 °C for 1 h. The boiled bacterial suspensions were cooled to room temperature, mixed with O83 antisera (SSI Diagnostica) in 96-well round-bottom microtitre plates. The plates were covered, incubated at 37 °C for 2 h. O83 serum agglutination was evaluated qualitatively by visual comparison with the negative control and was recorded as either positive or negative according to the presence or absence of visible agglutination.

### Growth *in vitro* of bacterial culture

Bacterial growth assay was performed as previously described [29]. OD at 660 nm was measured to assess bacterial growth.

### Antimicrobial killing assay

*In vitro* killing assay with polymyxin B was performed as described previously [52]. In brief, bacteria grown to the stationary growth phase were washed and resuspended in PBS. The diluted bacterial suspension at the density of 1–5×10^7^ c.f.u. ml^−1^ was treated with polymyxin B sulphate (0.4 µg ml^−1^; FUJIFILM Wako Pure Chemical Corporation) at 37 °C for 20 min. After the incubation, the mixture was plated on the LB medium, incubated at 37 °C overnight. The recovered c.f.u. was normalized for the original c.f.u. of the inoculum, thus yielding the bacterial ‘survival’ (in %).

### Epithelial cell invasion assay

HeLa cells were used as a well-established epithelial cell model for quantitative bacterial invasion assays because they provide a robust and reproducible system for assessing bacterial internalization. HeLa cells were maintained in Dulbecco’s modified Eagle’s medium supplemented with FBS under standard culture conditions. Briefly, monolayers of HeLa cells were infected with bacteria at an m.o.i. of 10 for 3 h at 37 °C with 5% CO_2_. Invasion ability was determined by a gentamicin protection method, where 50 µg ml^−1^ gentamicin was added to kill extracellular bacteria for determination of the numbers of invaded bacteria. Invasion efficiency was calculated based on the number of intracellular bacteria recovered from infected cells. Relative invasiveness was compared with that of LF82.

### RNA isolation from bacteria and reverse-transcription quantitative real-time PCR

Bacterial RNA was extracted from cultures using the Direct-zol RNA MiniPrep kit (Zymo Research) according to the manufacturer’s protocol. Reverse transcription was conducted using TaqMan Reverse Transcription reagents (Invitrogen). Quantitative PCR (qPCR) was performed with a CFX Opus 96 real-time PCR detection system (Bio-Rad), using SYBR Fast qPCR master mix (Kapa Biosystems) and the following primer sets: qLFfimA-FW (5′-AGGTAATGAGCTGACGCTGGA-3′) and qLFfimA-RV (5′-CAAAATAACGCGCCTGGAA-3′) for *fimA*; qLFfimH-FW (5′-CGGCTGTGATGTTTCTGCTC-3′) and qLFfimH-RV (5′-CCCCAGGTTTTGGCTTTTC-3′) for *fimH*; qLFfliC-FW (5′-ACTAGCGAAGGCACAGTAACAAAAG-3′) and qLFfliC-RV (5′-TAGTGGTGTTGTTCAGGTTGGTG-3′) for *fliC*; and qLFrpoD-FW (5′-TCTGATCACCGGCTTTGTTG-3′) and qLFrpoD-RV (5′-TCGTCTTCATCTTCGTCATCGT-3′) for *rpoD*. Relative transcript levels were normalized to the values for the *rpoD* gene and calculated by using 2^−∆*CT*^ method [53]. The housekeeping gene *rpoD* was used as an internal reference as in our previous studies [29, 30].

### Swimming motility assay

LB containing 0.3% agar was used to measure swimming motility as described previously [54]. In brief, a 5 µl aliquot at an OD_600_ of 0.6 was placed on a 0.3% agar LB plate and incubated at 37 °C for 16 h.

### Determination of *fimS* orientation by PCR and qPCR

The fimbrial phase was determined by *fimS* ON and OFF orientation as previously described [29]. Briefly, PCR was performed with genomic DNA (gDNA) extracted from LB cultures as the template and the following primer sets: FIMA (5′-GATGCGGTACGAACCTGTCC-3′) and INV (5′- GAGGTGATGTGAAA TTAATTTAC-3′) for the *fimS* ON orientation; FIME (5′-GCAGGCGGTTTCTTACGGGG-3′) and INV for the *fimS* OFF orientation. The amplified products were subjected to electrophoresis on a 2% agarose gel.

Quantification of the ON orientation of *fimS* was performed as previously described [29]. qPCR was performed with a CFX Opus 96 real-time PCR detection system (Bio-Rad) under standard cycling conditions for SYBR Fast qPCR master mix (Kapa Biosystems) using 10 ng of extracted gDNA and the FIMA and INV primer set. The relative abundance of ON orientation was normalized to the *rpoD* gene and calculated using the 2^−∆*CT*^ method [53]. For *fimS* orientation analysis, *rpoD* was used as an internal chromosomal reference to normalize the amount of template DNA.

### Yeast cell agglutination assay

Functional expression of type 1 fimbriae was evaluated by yeast cell agglutination assays, as previously described [55]. Briefly, commercial baker’s yeast was washed and resuspended in PBS (OD_600_ of 0.5). Likewise, bacteria grown at 37 °C in LB medium were washed and resuspended in PBS at an OD_600_ of 0.5. The bacterial suspension was mixed with an equal volume of the yeast cell suspension in each well of a 48-well plate. The plate was rocked gently on ice for 60 min. Yeast agglutination was evaluated semi-quantitatively by visual inspection and scored as +, ++ or +++, corresponding to weak, moderate and strong agglutination, respectively.

### Murine gut colonization experiment

C57BL/6 mice used in this study were housed under specific pathogen-free conditions at the animal experimental facilities of the School of Pharmacy, Kitasato University, or were purchased from Japan SLC. All animal protocols were reviewed and approved by the Kitasato University Institutional Animal Care and Use Committee (permit number: 24-8). Murine gut colonization assay was performed as previously described [28]. Briefly, 6- to 12-week-old mice were treated with a 2% (wt/vol) dextran sulphate sodium (DSS, molecular mass, 5,000 Da; FUJIFILM Wako Pure Chemical Corporation) in drinking water starting 7 days before infection and continuing until the end of the experiment. Furthermore, the mice were pretreated by oral gavage of streptomycin (25 mg per mouse) at 24 h before infection and were subsequently challenged orally with a 1 : 1 mixture of LF82 and the isogenic mutant or plasmid-transformed complementation strain (total 5×10^9^ c.f.u.). At least three mice were included in each experimental group. Mice were randomly assigned to the experimental groups. A total of ten male and seven female mice were used in the experiments comparing LF82 with the mutant strain (∆*rfaG::kan*) or the complemented strain (∆*rfaG* pLDΩKm2-*rfaG*). In addition, a total of 15 female mice were used in the experiments comparing LF82 with the complemented strains (∆*rfaG* pHSG575 and ∆*rfaG* pHSG-*fimA-H*). No obvious differences related to sex were noted during the study. In addition, because each competitive infection experiment was performed independently, the number of mice used varied slightly among the strain comparisons. To determine colonization levels of LF82 and the derivatives, faecal pellets, ileum tissue and colon tissue were collected and homogenized in sterile PBS using a TissueLyser device (Qiagen) for 2 min at a frequency of 25 Hz and plated at serial dilutions with PBS onto LB agar plates supplemented with ampicillin or kanamycin or chloramphenicol. After overnight incubation at 37 °C, colonies were counted. All strains were resistant to ampicillin. For the mouse colonization experiments, the ∆*rfaG::kan* mutant and the complemented strains (∆*rfaG* pLDΩKm2-*rfaG* or ∆*rfaG* pHSG-*fimA-H*) were additionally recovered on kanamycin- or chloramphenicol-containing agar plates, as appropriate, allowing differential enumeration of LF82 and the corresponding mutant or complemented strain. The c.f.u. of LF82 was calculated by subtracting the number of colonies recovered on kanamycin- or chloramphenicol-containing agar plates from the total number of colonies recovered on ampicillin-containing agar plates. Sample processing and c.f.u. enumeration were not performed in a blinded manner. The competitive index (CI) was determined by calculating the ratio of LF82 populations to their corresponding mutant and complementation derivatives and normalizing the ratio to the initial inoculum.

### Statistical analysis

Statistical tests were performed using GraphPad Prism v.10 for MacOS (GraphPad Software). Statistical significance (*P*<0.05) was determined by one-way ANOVA, followed by Dunnett’s multiple comparisons test, the one-sample t-test or the Mann–Whitney *U*-test. The statistical test used is described in the figure legends.

## Results

### Deletion of *rfaG* impairs agglutination with anti-O-antigen serum and outer membrane integrity in AIEC LF82

**Fig. 1.**
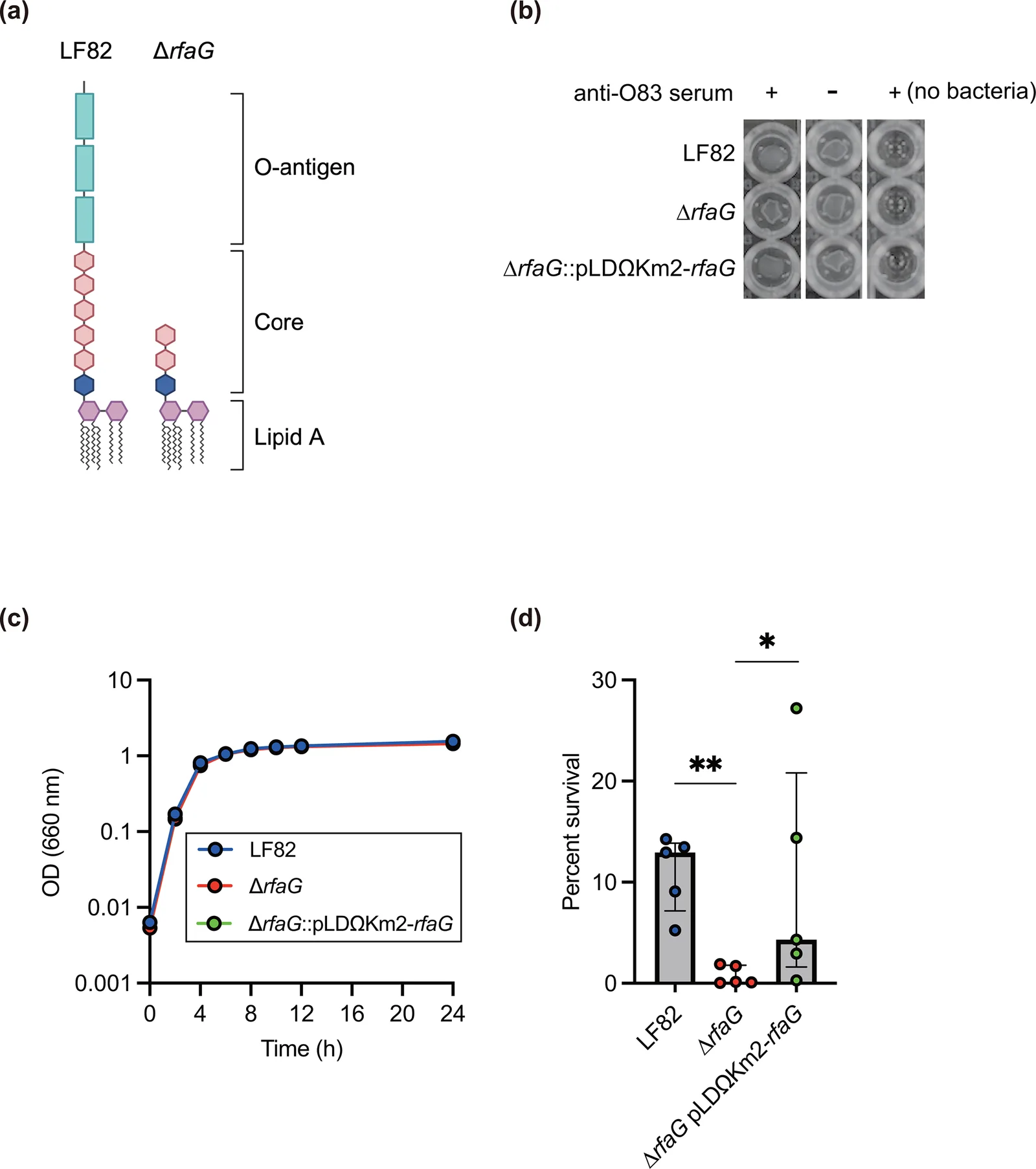
LF82 *rfaG* mutant exhibits defective LPS biogenesis, leading to reduced resistance to antimicrobial peptides. (**a**) LPS structure of LF82 and the ∆*rfaG* mutant. (**b**) Bacterial agglutination resulting from the interaction of O83 antisera with boiled bacterial suspension of LF82, the ∆*rfaG*, and the ∆*rfaG* pLDΩKm2-*rfaG*. Agglutination was evaluated visually. (**c**) Growth curves of LF82, the ∆*rfaG*, and the ∆*rfaG* pLDΩKm2-*rfaG*. Data points represent the means; *n*=3. Assays were done in three independent experiments. (**d**) Bacterial survival against polymyxin B. LF82, the ∆*rfaG* and the ∆*rfaG* pLDΩKm2-*rfaG* were exposed to 0.4 µg ml^−1^ polymyxin B for 20 min, and bacterial survival was quantified by dilution plating on LB agar. Percentage of c.f.u. values remaining after exposure to polymyxin B. *n* is indicated by the number of dots. Assays were performed in three independent experiments. Bars, median with interquartile range. ns, not significant; ^*^P<0.05; ^**^P<0.01; two-tailed Mann–Whitney *U*-test.

To investigate the role of LPS in AIEC pathogenesis, we generated an isogenic *rfaG* deletion mutant in the AIEC strain LF82. Because *rfaG* encodes a glycosyltransferase involved in LPS core biosynthesis, deletion of *rfaG* was expected to disrupt O-antigen production and generate a rough LPS phenotype [6, 56] (Fig. 1a). To evaluate O-antigen expression, we performed bacterial agglutination assays using O83 antisera. Although the parental LF82 strain exhibited strong agglutination, the ∆*rfaG* mutant failed to agglutinate in the presence of O83 antisera (Fig. 1b). Complementation of the ∆*rfaG* mutant with chromosomally integrated *rfaG* restored agglutination activity.

We next examined the growth rates by measuring the OD at 600 nm (OD_600_) of bacterial growth in the LB medium to clarify whether the lack of O-antigen could affect bacterial growth. The OD_600_ values of the ∆*rfaG* culture were similar to those of LF82 and the ∆*rfaG*::pLDΩKm2-*rfaG* (Fig. 1c), indicating that deletion of the *rfaG* gene does not influence the bacterial growth of LF82.

LPS is a constituent of the outer membrane, thereby contributing to protection of bacterial cells against antimicrobials, as evidenced by previous results showing that *rfaG* deletion of *E. coli* leads to outer membrane instability due to enhanced penetration across the outer membrane [57, 58]. Thus, to further confirm disruption of LPS integrity, we examined susceptibility to polymyxin B, an antimicrobial peptide that targets the outer membrane of Gram-negative bacteria. LF82 and the ∆*rfaG* were incubated with polymyxin B for 1 h, and bacterial survival was quantified by dilution plating. As expected, the ∆*rfaG* mutant exhibited increased susceptibility to polymyxin B compared with the parental LF82 strain (Fig. 1d), consistent with defective outer membrane integrity caused by impaired LPS biogenesis. Complementation with *rfaG* restored polymyxin B resistance to levels comparable to those of the parental strain. Collectively, these results indicate that deletion of *rfaG* leads to impaired agglutination with anti-O-antigen antiserum and a defective outer membrane barrier. In accordance with previous studies, *rfaG* may be required for proper LPS biosynthesis and O-antigen production in AIEC strain LF82.

### Deletion of *rfaG* impairs epithelial invasion by AIEC LF82

**Fig. 2.**
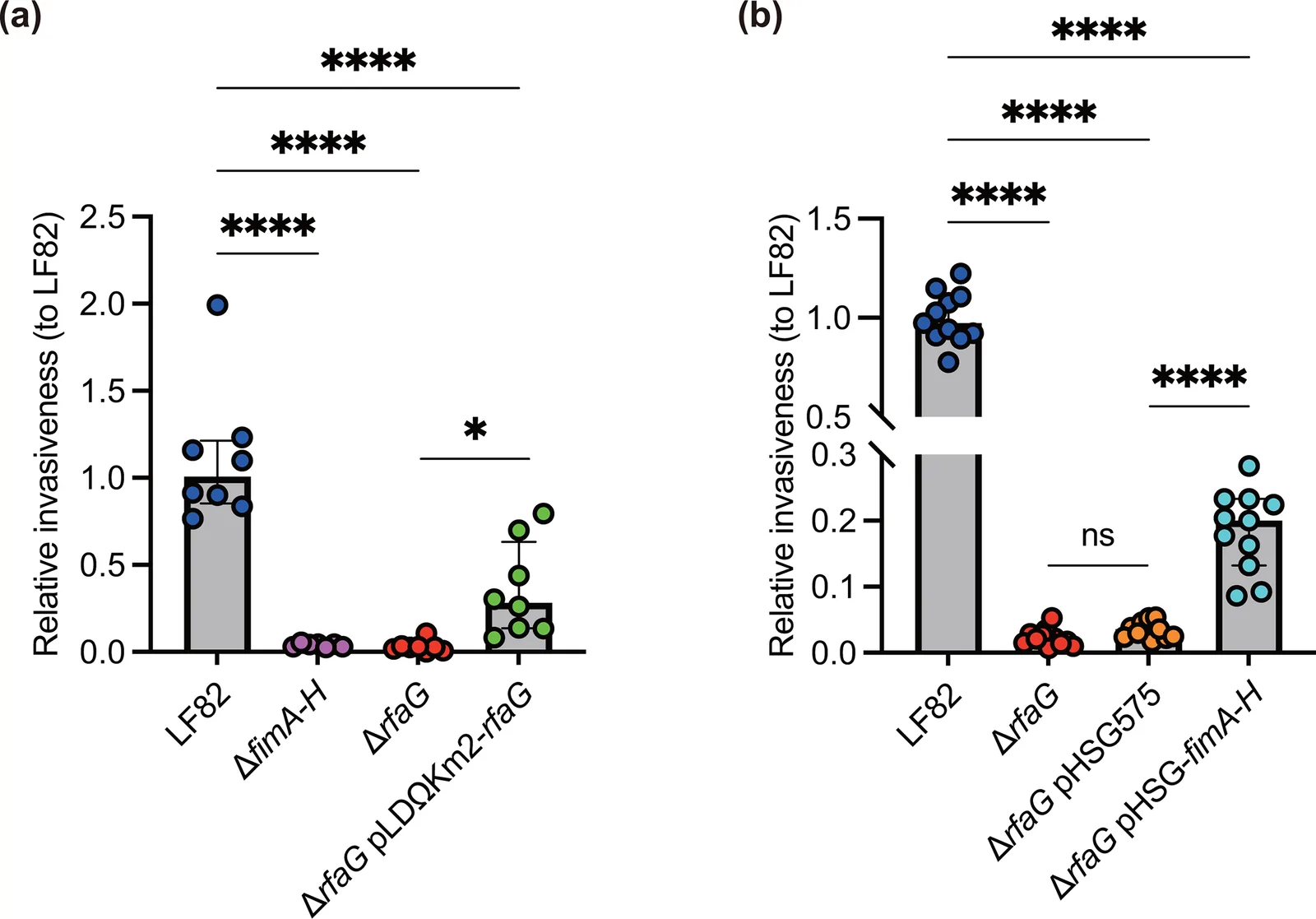
LF82 *rfaG* mutant has reduced invasiveness in HeLa cells, which was restored by introduction of plasmids encoding *rfaG* or the *fim* operon. (**a**) HeLa cells were infected with LF82, the ∆*fimA-H*, the ∆*rfaG* and the ∆*rfaG* pLDΩKm2-*rfaG*, and their ability to invade HeLa cells was determined. (**b**) The ability of LF82, the ∆*rfaG*, the ∆*rfaG* pHSG575 and ∆*rfaG* pHSG-*fimA-H* to invade HeLa cells was determined. Quantified invasiveness was determined by defining that of LF82 as 1. *n* is indicated by the number of dots. Assays were done at least in three independent experiments. Bars, median with interquartile range. ns, not significant; ^*^P<0.05; ^****^P<0.0001; one-way ANOVA followed by Dunnett’s multiple comparisons test.

Adherence to and subsequent invasion of host epithelial cells in the intestinal tract are important steps in LF82 pathogenesis [59]. Furthermore, type 1 fimbriae are major virulence determinants required for epithelial adhesion and invasion by AIEC [22]. Thus, we next tested the ability of the ∆*rfaG* to invade epithelial cells using an *in vitro* assay. HeLa cells were infected with LF82, the ∆*rfaG* and the type 1 fimbriae-deficient ∆*fimA-H* mutant. The invasion efficiencies were determined by quantifying the intracellular bacteria (see Methods). LF82 efficiently invaded HeLa cells, whereas a severe invasion defect was observed in the ∆*fimA-H* mutant (Fig. 2a). The results verified the critical role of type 1 fimbriae in cell invasion by LF82, in line with previous data showing that *fim*-encoded type 1 fimbriae-mediated adherence plays a critical role in epithelial cell invasion process [22, 60]. In contrast, the ∆*rfaG* mutant exhibited reduced invasion, which was partially restored in the ∆*rfaG*::pLDΩKm2-*rfaG* (Fig. 2a).

Next, we investigated whether type 1 fimbriae are implicated in impaired invasion of the ∆*rfaG*. To this end, we employed the ∆*rfaG* transformant with a plasmid encoding the *fimA-H* operon genes, in which transcription of the *fim* genes is induced from the plasmid-based *lac* promoter, resulting in induced expression of type 1 fimbriae [29]. Introduction of the control plasmid pHSG575 did not affect the capacity to invade HeLa cells, whereas the transformed strain invaded more efficiently compared to the control plasmid-transformed strain (Fig. 2b). The results raise the possibility that the *rfaG*-dependent internalization phenotype, at least in part, is attributable to type 1 fimbrial expression. Collectively, these results indicate that *rfaG* contributes to AIEC LF82 internalization, which is determined by both bacterial adherence to and invasion into cells.

### *rfaG* is required for expression of type 1 fimbrial genes in AIEC LF82

**Fig. 3.**
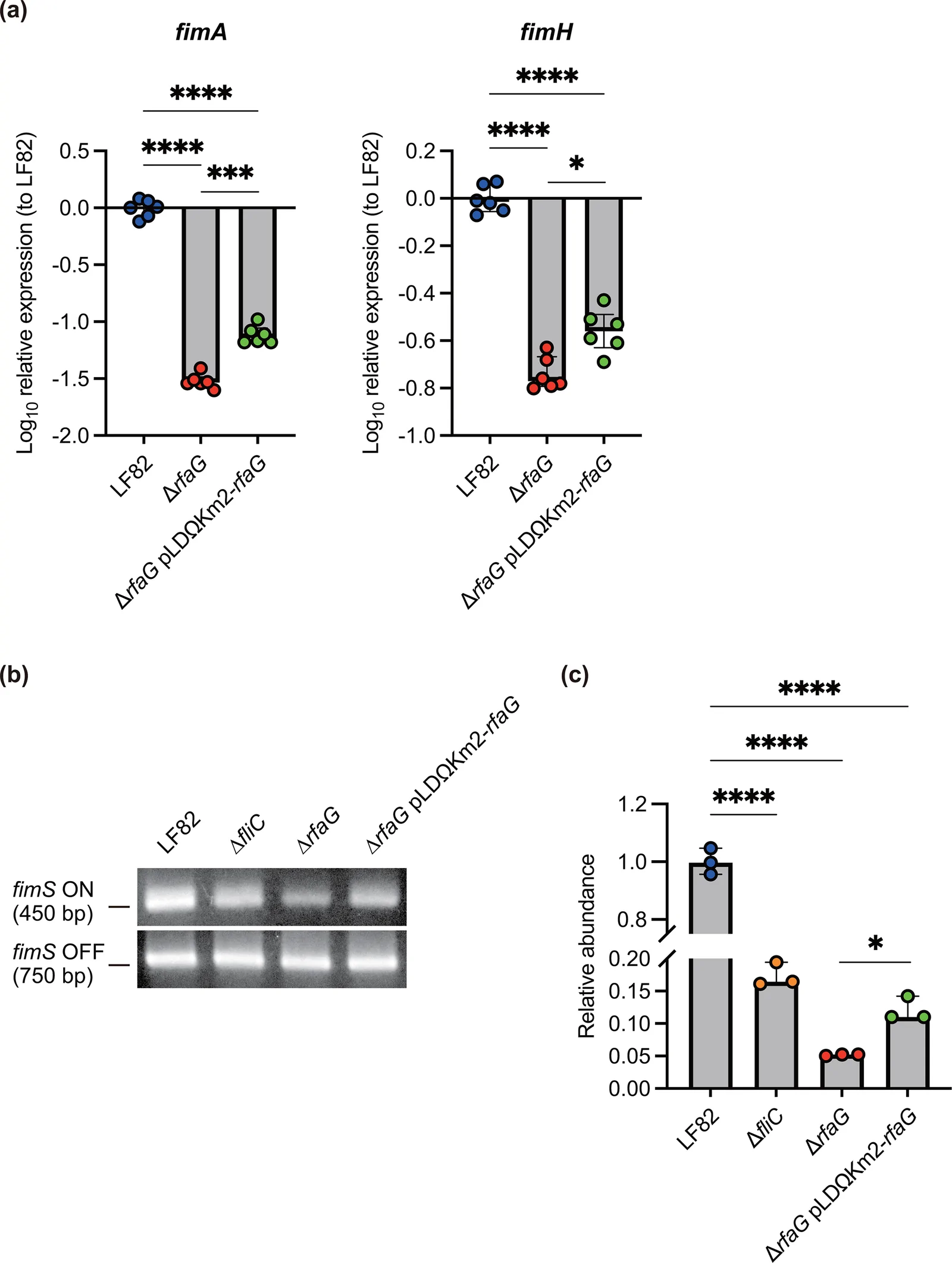
LF82 *rfaG* mutant exhibits reduced expression of fimbrial genes. (**a**) Transcript levels of *fimA* and *fimH* were determined by RT-qPCR. Data were normalized to *rpoD* expression levels. *n* is indicated by the number of dots. Assays were done in two independent experiments. Bars, median with interquartile range. ^*^P<0.05; ^***^P<0.001; ^****^P<0.0001; one-way ANOVA followed by Dunnett’s multiple comparisons test. (**b**) The orientation of invertible *fimS* DNA element was determined by PCR as previously described [29]. Similar results were obtained in three independent experiments. (**c**) ON-phase population was determined by qPCR analysis as previously described [29]. Relative abundance of ON orientation was normalized to the *rpoD* gene. *n* is indicated by the number of dots. Assays were done in three independent experiments. Bars, median with interquartile range. ^*^P<0.05; ^****^P<0.0001; one-way ANOVA followed by Dunnett’s multiple comparisons test.

To determine whether the invasion defect of the ∆*rfaG* mutant was associated with altered type 1 fimbriae expression, we first analysed transcription of the fimbrial genes by reverse-transcription quantitative real-time PCR (qRT-PCR). Expression levels of *fimA* and *fimH* were significantly reduced in the ∆*rfaG* mutant compared with the parental LF82 strain (Fig. 3a). Complementation with *rfaG* partially restored transcription of these genes. These results indicate that a mutation of the *rfaG* leads to reduced transcriptional activity of the fimbrial genes.

Type 1 fimbriae expression is controlled by phase variation mediated through inversion of the *fimS* promoter region located upstream of the *fim* operon, thereby generating either a piliated ON phase or a non-piliated OFF phase [32]. To investigate whether *rfaG*-dependent LPS biogenesis influences phase variation, we next analysed *fimS* orientation using a PCR-based assay (see Methods). LF82 contained mixed ON- and OFF-phase populations, whereas the ∆*fliC* mutant exhibited a reduced ON-phase population, consistent with previous findings that flagellar deficiency alters *fimS* phase variation [60]. Likewise, the ∆*rfaG* mutant showed a marked reduction in the ON-phase population compared with LF82 (Fig. 3b). Partial restoration of the ON-phase population was observed in the complemented strain ∆*rfaG*::pLDΩKm2-*rfaG*. In agreement with these findings, qPCR analysis demonstrated that deletion of *rfaG* significantly decreased the abundance of the ON-phase population (Fig. 3c). These results suggest that impaired fimbrial gene expression in the ∆*rfaG* mutant is associated, at least in part, with altered *fimS* phase variation.

### Deletion of *rfaG* impairs flagellar motility of AIEC LF82 through reduced *fliC* expression

**Fig. 4.**
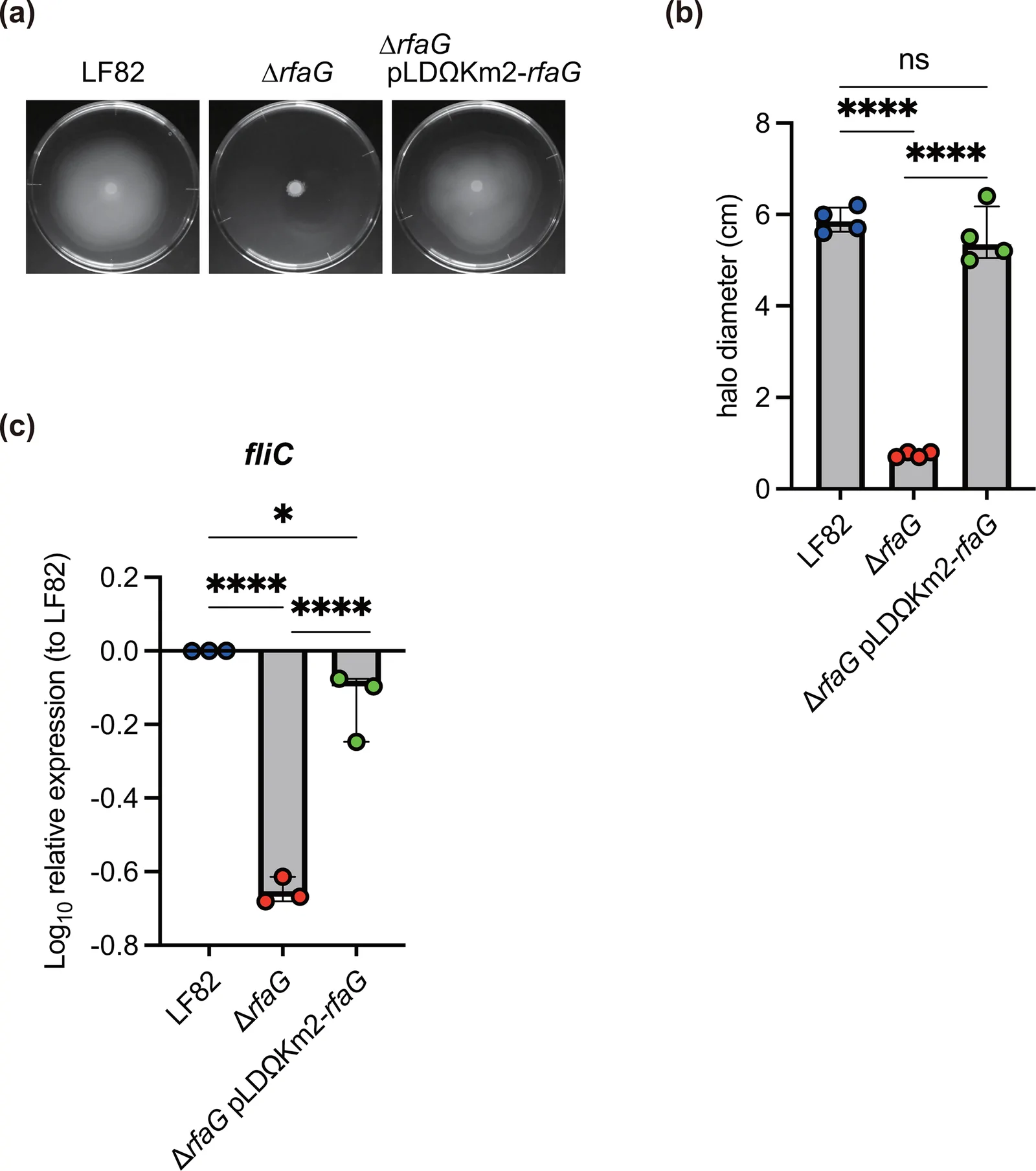
LF82 *rfaG* mutant exhibits reduced motility and decreased expression of the flagellin gene *fliC*. (**a**) Motility on soft agar LB plate. LF82, the ∆*rfaG* and ∆*rfaG* pLDΩKm2-*rfaG* were spotted on LB with 0.3% agar and incubated at 37 °C for 16 h. Similar results were obtained in two independent experiments. (**b**) The halo of the motility assay was measured. Assays were performed in two independent experiments. (**c**) Transcript levels of *fliC* were determined by RT-qPCR. Data were normalized to *rpoD* expression levels. *n* is indicated by the number of dots. Assays were done in three independent experiments. Bars, median with interquartile range. ns, not significant; ^*^P<0.05; ^****^P<0.0001; one-way ANOVA followed by Dunnett’s multiple comparisons test.

Because flagellar function has previously been implicated in regulation of type 1 fimbriae expression in AIEC LF82 [60], we next examined whether deletion of *rfaG* affects bacterial motility. The *∆rfaG* mutant exhibited markedly reduced swimming motility on soft LB agar (0.3% agar) compared with the parent LF82 strain. This motility defect was restored in the complemented strain ∆*rfaG*::pLDΩKm2-*rfaG* (Fig. 4a and b).

To investigate the molecular basis of the impaired motility phenotype, we next quantified transcription of the flagellin gene *fliC* by RT-qPCR. Expression of *fliC* was significantly reduced in the ∆*rfaG* mutant compared with the parent LF82 strain (Fig. 4c). Complementation with *rfaG* partially restored *fliC* expression. These findings indicate that deletion of *rfaG* impairs flagellar motility, at least in part, through reduced expression of *fliC*.

Previous studies demonstrated that loss of *fliC* reduces type 1 fimbriae expression in AIEC LF82 [60]. Together with our observation that the ∆*rfaG* mutant exhibits reduced *fliC*, *fimA* and *fimH* expression, these findings raise the possibility that impaired flagellar function contributes to the reduced fimbrial gene expression observed in the ∆*rfaG* mutant.

### *rfaG* is required for type 1 fimbriae expression in AIEC LF82

**Fig. 5.**
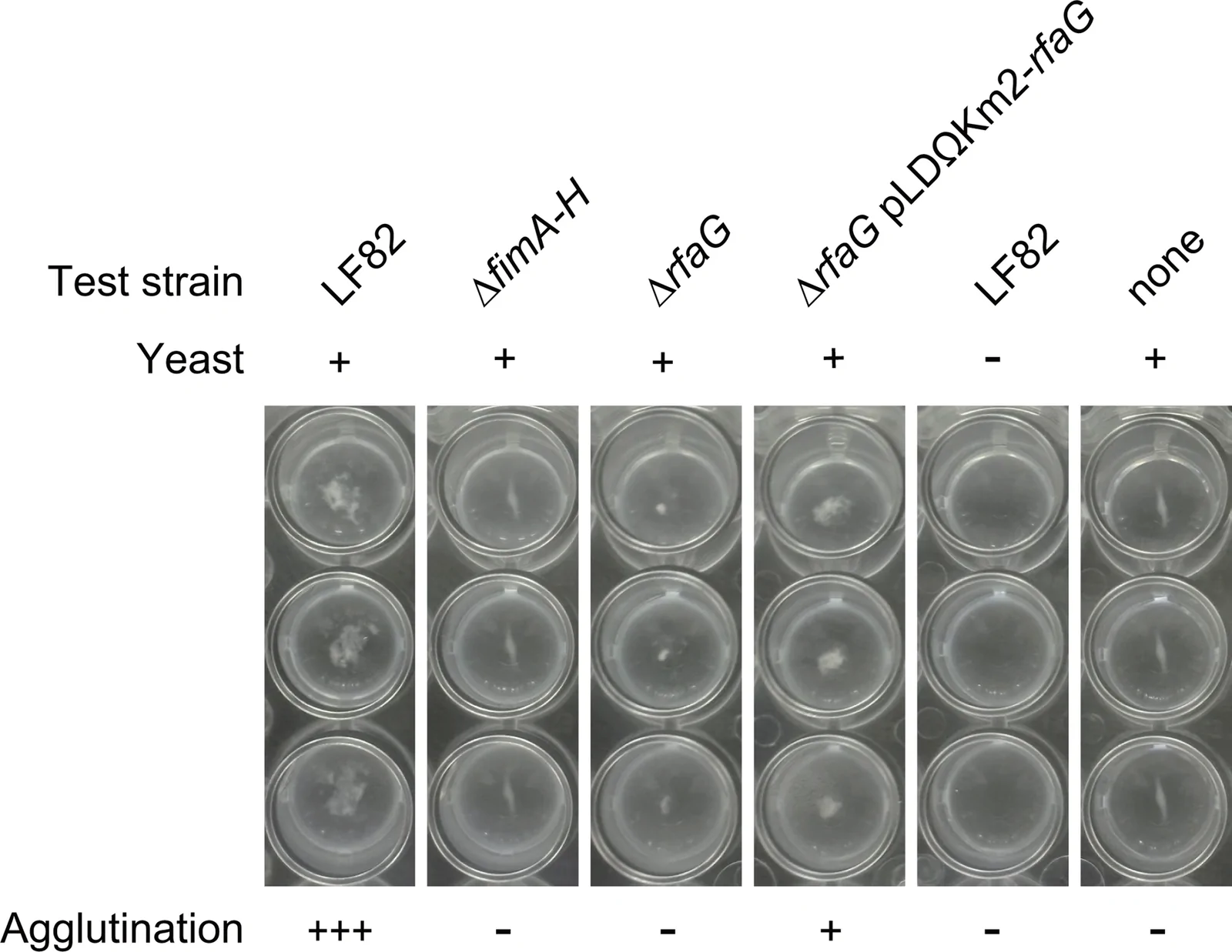
LF82 *rfaG* mutant exhibits reduced yeast cell agglutination. Yeast cell agglutination resulting from interactions with bacterial suspensions of LF82, the ∆*fimA-H*, the ∆*rfaG* and the ∆*rfaG* pLDΩKm2-*rfaG* was evaluated. Yeast cell agglutination was scored visually as strong (+++), weak (+) or absent (−). Similar results were obtained in three independent experiments.

We examined the functional expression of type 1 fimbriae using yeast cell agglutination assays (see Methods). Surface-assembled type 1 fimbriae can bind to mannose residues on the yeast cell surface and consequently lead to agglutination of yeast cells [55]. Thus, the yeast cell agglutination assay is suitable for the semi-quantitative assessment of type 1 fimbrial-mediated binding, as used in our previous studies [28–30]. LF82 induced agglutination, whereas no agglutination was observed in the ∆*fimA-H* mutant (Fig. 5). In contrast, the mixture of ∆*rfaG* cells and yeast cells resulted in smaller clumps compared with the LF82-induced clumps. Furthermore, the ∆*rfaG*::pLDΩKm2-*rfaG* induced the agglutination similar to LF82. These results indicate that *rfaG* is required for the transcriptional and functional expression of type 1 fimbriae in AIEC LF82.

### Deletion *rfaG* impairs competitive intestinal colonization in a type 1 fimbriae expression-dependent manner

**Fig. 6.**
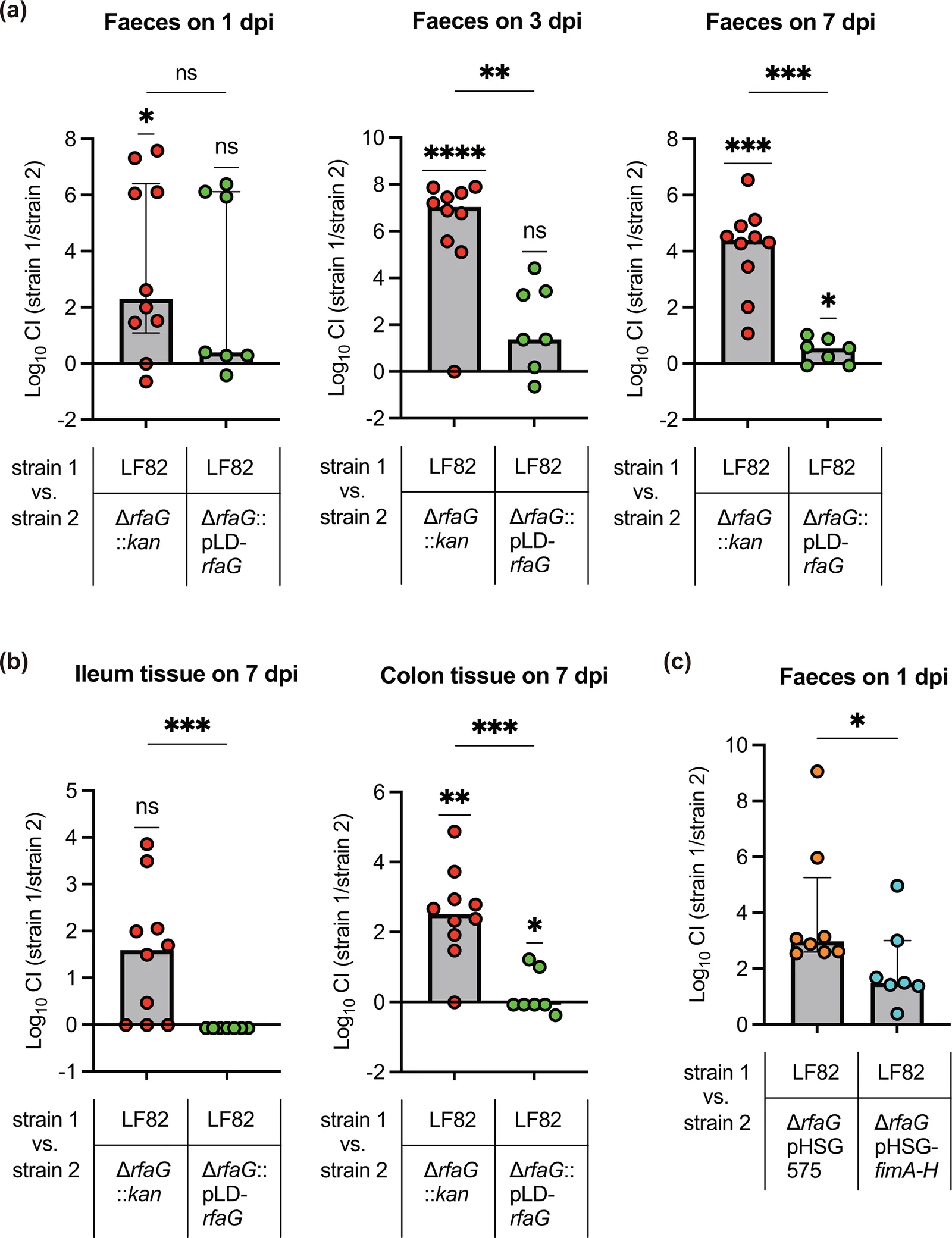
LF82 *rfaG* mutant is impaired in competitive colonization in a DSS mouse model. (**a, b**) DSS- and streptomycin-pretreated C57BL/6 mice were infected via oral gavage with a 1:1 mixture of LF82 and the ∆*rfaG::kan* mutant or LF82 and the ∆*rfaG* pLDΩKm2-*rfaG* complemented strain. (**a**) Faecal CI values for faeces on days 1, 3 and 7 p.i. (**b**) CI values for the ileum and colon tissues on day 7 p.i. (**c**) DSS- and streptomycin-pretreated C57BL/6 mice were infected via oral gavage with a 1:1 mixture of LF82 and the ∆*rfaG::kan* pHSG575 or LF82 and the ∆*rfaG* pHSG-*fimA-H*. Faecal CI values for faeces on day 1 p.i. *n* is indicated by the number of dots. Assays were performed at least in two independent experiments. dpi, days postinfection. Bars, median with interquartile range. ns, not significant; ^*^P<0.05; ^**^P<0.01; ^***^P<0.001; ^****^P<0.0001; one sample t-test and two-tailed Mann–Whitney *U*-test.

Finally, to determine whether impaired type 1 fimbriae expression contributes to the colonization defect of the ∆*rfaG* mutant *in vivo*, we performed competitive infection experiments in mice. DSS- and streptomycin-treated C57BL/6 mice were orally infected with an equal mixture of LF82 and a mutant strain in which the *rfaG* gene is replaced with a kanamycin resistance cassette (∆*rfaG::kan*). Like the ∆*rfaG* mutant, the ∆*rfaG::kan* mutant also exhibited reduced epithelial invasion, confirming that the two mutant strains display comparable phenotypes apart from the kanamycin resistance cassette. We determined the bacterial loads in faeces on days 1, 3 and 7 post-infection (p.i.) by dilution selective plating (see Methods) and calculated the CI values to evaluate the abilities of LF82 and the ∆*rfaG::kan* to colonize the gut competitively. The CI values from the mouse infection experiments showed that the ∆*rfaG::kan* exhibited reduced competitive fitness in the gut, indicating that the ∆*rfaG::kan* mutant was outcompeted by LF82 on days 1, 3 and 7 p.i. (Fig. 6a). In contrast, the CI values of LF82 and the ∆*rfaG* pLDΩKm2-*rfaG* showed that the competitive colonization of the ∆*rfaG* pLDΩKm2-*rfaG* was significantly restored on days 3 and 7 p.i. (Fig. 6a). Furthermore, we analysed the CI values of ileum tissue and colon tissue on day 7 p.i. Likewise, the competitive colonization levels of the ∆*rfaG::kan* in both tissues were reduced in comparison with LF82, whereas the ∆*rfaG* pLDΩKm2-*rfaG* were restored (Fig. 6b).

To determine whether reduced type 1 fimbriae expression contributes to the colonization defect associated with *rfaG* deletion, we performed competitive infection experiments using the ∆*rfaG* carrying a plasmid expressing the fimbrial operon (pHSG-*fimA-H*). Expression of the fimbrial operon significantly improved the competitive fitness of the ∆*rfaG* mutant on day 1 p.i. compared with the vector control strain carrying pHSG575 (Fig. 6c). These findings indicate that impaired type 1 fimbriae expression is a major contributor to the colonization defect of the ∆*rfaG* mutant at the early stage of infection. In contrast, the *rfaG*-dependent persistent colonization may involve mechanisms independent of type 1 fimbrial expression. Collectively, the results support a model in which *rfaG* promotes intestinal colonization of AIEC LF82 by maintaining type 1 fimbriae expression, particularly during the early stage of infection.

## Discussion

**Fig. 7.**
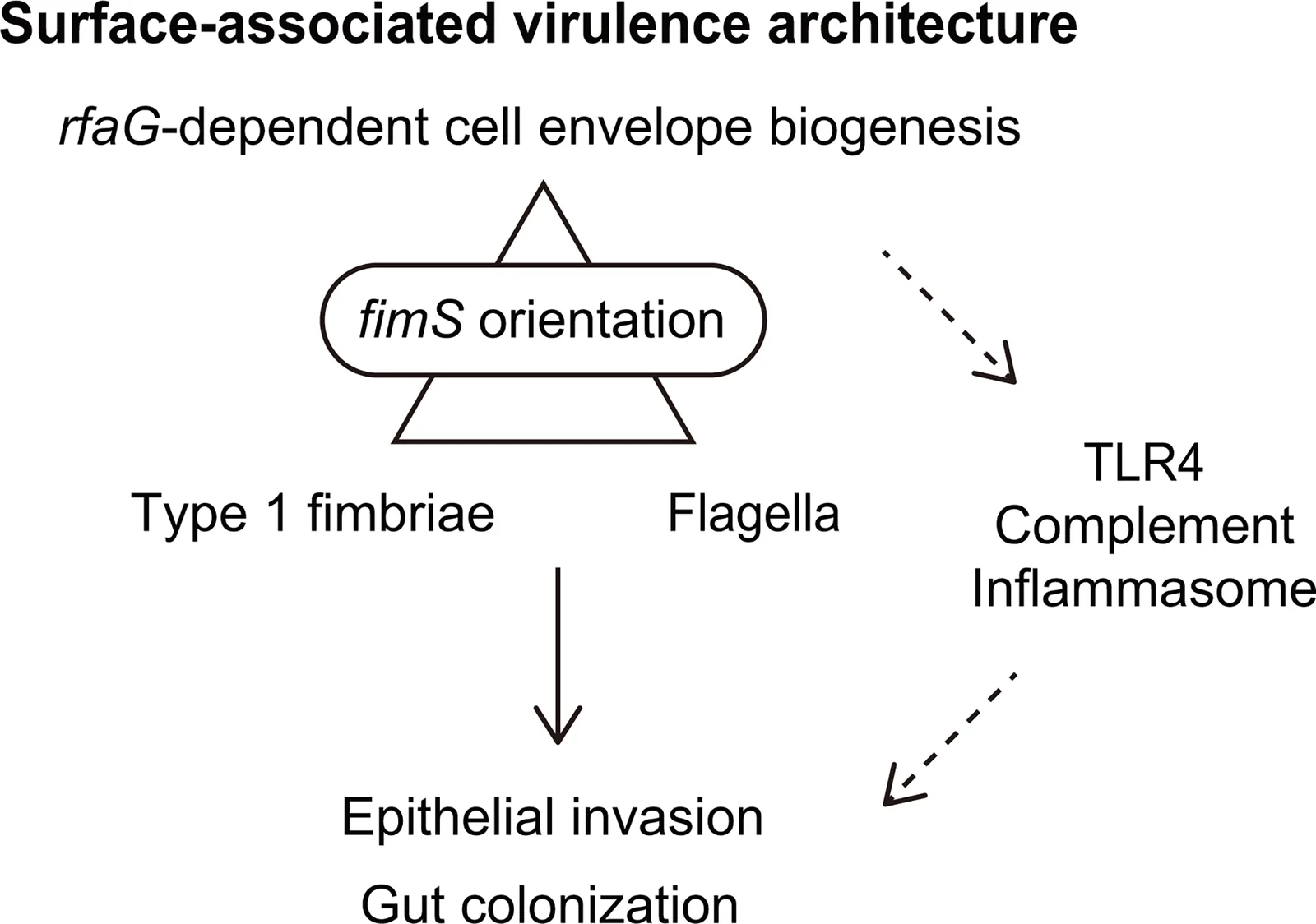
Proposed working model: surface-associated virulence architecture of AIEC LF82. *rfaG*-dependent cell envelope biogenesis, flagella and type 1 fimbriae collectively form a surface-associated virulence architecture that contributes to both host inflammatory responses and bacterial colonization. *rfaG*-dependent cell envelope biogenesis promotes inflammatory responses through activation of TLR4 signalling, the complement system and the inflammasome [42, 43, 61]. In parallel, *rfaG*-dependent cell envelope biogenesis may influence *fimS* orientation through flagella-associated regulatory pathways, thereby contributing to the expression of type 1 fimbriae and facilitating epithelial invasion and intestinal colonization. The present study suggests that these surface-associated structures are functionally interconnected and may collectively promote AIEC fitness within the intestinal environment. Solid arrows indicate associations supported by the present study, whereas dashed arrows indicate regulatory relationships proposed on the basis of previous studies.

The present study aimed to investigate the role of LPS in the pathogenesis of the CD-associated AIEC strain LF82. Previous studies demonstrated that AIEC LPS contributes to intestinal inflammation through activation of TLR4 signalling and modulation of the complement system [42, 61]. Furthermore, AIEC LPS-mediated inflammasome activation in macrophages drives intestinal inflammation, which is intimately linked to AIEC virulence [43]. Here, we identified an additional and previously unrecognized role of AIEC LPS in epithelial invasion and intestinal colonization through its effects on type 1 fimbriae expression. Together with previous findings showing the role of LPS in AIEC–host interactions [42, 43, 61], our results support a working model in which AIEC *rfaG*-dependent cell envelope biogenesis, flagella and type 1 fimbriae form a coordinated surface-associated virulence architecture that contributes to both host inflammatory responses (previous studies [42, 43, 61]) and bacterial colonization of the intestinal tract (this study) and provide a framework for future investigation of the regulatory relationships among these surface-associated structures (Fig. 7).

In the present study, deletion of *rfaG* resulted in reduced expression of type 1 fimbriae and impaired epithelial invasion by LF82. Furthermore, the ∆*rfaG* mutant exhibited a marked defect in intestinal colonization in DSS-treated mice. Importantly, inducible expression of type 1 fimbriae rescued the colonization defect of the mutant strain, indicating that LPS-dependent regulation of type 1 fimbriae contributes substantially to intestinal colonization by LF82. Notably, however, this rescue effect was largely restricted to the early stage of infection (day 1 p.i.), consistent with our previous findings indicating that type 1 fimbriae expression contributes to early-stage intestinal colonization but not persistent colonization in the murine intestinal colonization model [28]. This observation suggests that *rfaG*-dependent intestinal colonization cannot be explained solely by type 1 fimbriae expression and raises the possibility that additional LPS-associated factors contribute to long-term persistence of LF82 within the intestinal tract. On the other hand, it is known that mutations in *rfa* genes in *E. coli*, including *rfaG*, exert pleiotropic effects on cell envelope physiology, including impairment of the outer membrane barrier, particularly because the presence of phosphoryl substituents on the LPS core heptose residues is essential for membrane stability and structural cohesion [46, 62]. Notably, a previous study demonstrated that impairment of outer membrane barrier integrity in AIEC causes reduced persistent intestinal colonization [63]. Therefore, it is reasonable to propose that the *rfaG*-dependent defect in persistent colonization may, at least in part, reflect impaired maintenance of LPS-associated outer membrane integrity rather than altered type 1 fimbrial expression alone.

LPS is one of the best-characterized pathogen-associated molecular patterns recognized by TLR4 and is therefore a potent inducer of inflammatory responses. In AIEC-associated CD, persistent intestinal colonization by AIEC contributes to chronic inflammation through LPS-mediated activation of mucosal immune pathways [39]. AIEC colonization also alters the composition of the gut microbiota and increases luminal LPS levels [40]. Conversely, AIEC can partially evade innate immune responses by modulating microRNA expression, thereby suppressing autophagy and altering LPS–TLR4 signalling [42, 64]. Because intestinal inflammation is tightly linked to CD pathology, these findings reinforce the importance of LPS as a virulence-associated factor during persistent colonization of AIEC LF82.

Beyond its established role in inflammation, our findings demonstrate that the *rfaG*-dependent cell envelope biogenesis contributes to virulence-associated phenotypes, including epithelial cell invasion and intestinal colonization, through regulation of type 1 fimbriae expression. Type 1 fimbriae are major virulence determinants required for intestinal colonization and induction of gut inflammation by AIEC [22, 24, 25]. Expression of type 1 fimbriae is tightly controlled by multiple regulatory pathways that enable pathogenic bacteria to coordinate fimbrial expression in response to environmental conditions [65]. In pathogenic *E. coli*, including uropathogenic *E. coli*, fimbrial expression is influenced by diverse environmental and physiological cues, such as carbon metabolism, osmolarity, growth phase and pH [66, 67]. Similarly, previous studies demonstrated that AIEC modulates type 1 fimbriae expression in response to envelope stress and microbiota-derived metabolites [28, 29].

Interestingly, previous studies demonstrated that flagellar deficiency reduces type 1 fimbriae expression in AIEC [60], indicating the possible interplay between flagella and fimbrial expression. Consistent with this notion, we found that the *∆rfaG* mutant exhibited impaired swimming motility accompanied by reduced expression of the flagellin gene *fliC*. Furthermore, the ∆*rfaG* mutant displayed a reduced ON-phase orientation of *fimS*, indicating altered phase variation. Together, these findings raise the possibility that *rfaG*-dependent cell envelope biogenesis influences fimbrial expression, at least in part, through flagella-associated regulatory pathways and/or modulation of *fimS* phase variation. However, the present study does not establish the regulatory hierarchy among these phenotypes. Importantly, our findings may expand the flagella–fimbrial expression regulatory system to include *rfaG*-dependent cell envelope biogenesis, providing a working hypothesis that bacterial surface structures may coordinately regulate fimbrial expression.

Although chromosomal complementation of *rfaG* restored several mutant phenotypes, the restoration was incomplete in some assays. Because the complemented strain carries a single chromosomally integrated copy of *rfaG*, we cannot exclude the possibility that *rfaG* expression differed from that of the parental LF82 strain. Such differences may have contributed to the incomplete restoration of these phenotypes.

Although HeLa cells are widely used for bacterial invasion assays, they do not fully reproduce the intestinal epithelial environment. Therefore, future studies using intestinal epithelial cell lines or intestinal organoid models will be useful to further validate the role of *rfaG* in AIEC pathogenesis.

Importantly, our findings identify LPS as a previously unrecognized component of this regulatory network. Epithelial invasion by AIEC LF82 depends not only on type 1 fimbriae but also on flagella and outer membrane proteins [22, 60, 68]. Collectively, these observations support a model in which multiple bacterial surface-associated structures may cooperatively form a ‘surface-associated virulence architecture’ that governs epithelial invasion and gut colonization. This architecture comprises LPS, flagella and type 1 fimbriae, which collectively facilitate bacterial adaptation to the intestinal environment and promote AIEC pathogenesis. However, the underlying regulatory mechanism remains unsolved. Previous work showed that deletion of *rfaG* activates the Rcs envelope stress response, likely by sensing defects in LPS core biogenesis [44]. This signalling pathway negatively regulates the *flhDC* operon, which is required for flagellar expression [69], and reduced *flhDC* expression has also been reported following *fliC* deletion [60]. Therefore, rather than proposing a direct regulatory role for *rfaG*, we speculate that perturbation of *rfaG*-dependent cell envelope biogenesis induces an envelope stress response that subsequently reduces *flhDC* expression, leading to impaired flagellar expression. Given the coordinated regulation between flagella and type 1 fimbriae, this mechanism may indirectly contribute to the reduced expression of type 1 fimbriae observed in the ∆*rfaG* mutant (Fig. 7).

Because *fimS* orientation is controlled by multiple environmental and envelope-associated regulatory pathways, disruption of LPS biogenesis may alter the regulatory network governing *fim* phase switching through changes in outer membrane architecture.

Although disruption of *rfaG* represents an artificial genetic perturbation of LPS biosynthesis, alterations in LPS structure have been reported during the within-host evolution of enteric pathogens and during adaptation to selective pressures such as bacteriophage predation [70, 71]. These observations indicate that perturbations in LPS biosynthesis can occur under biologically relevant conditions, although such alterations are frequently associated with fitness trade-offs. Therefore, our findings should be interpreted as demonstrating the consequences of perturbing *rfaG*-dependent cell envelope biogenesis, rather than suggesting that *rfaG* itself functions as a physiological regulator of type 1 fimbrial expression.

Several limitations of this study should be acknowledged. First, all experiments were performed using the prototype AIEC strain LF82. Therefore, further studies using genetically diverse clinical AIEC isolates will be required to determine whether the role of *rfaG* in regulating type 1 fimbrial expression and intestinal colonization is conserved among AIEC strains. Second, although disruption of *rfaG* altered *fimS* orientation and reduced type 1 fimbrial expression, the molecular mechanism linking *rfaG*-dependent cell envelope biogenesis to *fimS* regulation remains to be elucidated. In addition, while disruption of *rfaG* likely affects cell envelope architecture, the structural alterations were not directly characterized in the present study. Finally, intestinal colonization was evaluated using a streptomycin-pretreated DSS mouse model, which facilitates AIEC colonization but does not fully recapitulate the complex intestinal environment associated with CD. Addressing these limitations in future studies will provide a more comprehensive understanding of the contribution of *rfaG*-dependent cell envelope biogenesis to AIEC pathogenesis.

In conclusion, we propose that the surface-associated virulence architecture coordinates the transition from epithelial interaction to persistent intestinal colonization. Future studies should clarify the molecular mechanisms linking *rfaG*-dependent cell envelope biogenesis to fimbrial regulation. Such studies may facilitate the development of novel therapeutic strategies for AIEC-associated CD.

## Abbreviations

AIEC: adherent-invasive *Escherichia coli*
CD: Crohn’s disease
CI: competitive index
DSS: dextran sulphate sodium
gDNA: genomic DNA
LB: Luria–Bertani
p.i.: post-infection
qPCR: quantitative polymerase chain reaction
RT-qPCR: reverse transcription and quantitative polymerase chain reaction
TLR4: Toll-like receptor 4
